# Suitability of frozen cell pellets from cytology specimens for the Amoy 9‐in‐1 assay in patients with non‐small cell lung cancer

**DOI:** 10.1111/1759-7714.15382

**Published:** 2024-06-19

**Authors:** Hiroaki Kodama, Haruyasu Murakami, Nobuaki Mamesaya, Haruki Kobayashi, Shota Omori, Kazushige Wakuda, Ryo Ko, Akira Ono, Hirotsugu Kenmotsu, Tateaki Naito, Shingo Matsumoto, Koichi Goto, Tetsuo Shimizu, Yasuhiro Gon, Toshiaki Takahashi

**Affiliations:** ^1^ Division of Thoracic Oncology Shizuoka Cancer Center Shizuoka Japan; ^2^ Respiratory Medicine and Infectious Diseases Oita University Faculty of Medicine Oita Japan; ^3^ Department of Thoracic Oncology National Cancer Center Hospital East Kashiwa Japan; ^4^ Division of Respiratory Medicine, Department of Internal Medicine Nihon University School of Medicine Tokyo Japan

**Keywords:** cell pellet, cytology specimen, non‐small cell lung cancer, oncogenic driver alteration, real‐time polymerase chain reaction

## Abstract

**Background:**

The AmoyDx Pan lung cancer PCR panel (AmoyDx PLC panel) has been approved as a companion diagnostic tool for multiple anticancer agents in patients with non‐small cell lung cancer (NSCLC). However, the suitability of cytology specimens as samples for the AmoyDx PLC panel remains unclear. We evaluated the performance of frozen cell pellets from cytology specimens (FCPs) in the Amoy 9‐in‐1 assay, a preapproval assay of the AmoyDx PLC panel.

**Methods:**

We retrospectively collected data of NSCLC patients enrolled in LC‐SCRUM‐Asia from the Shizuoka Cancer Center between September 2019 and May 2021.

**Results:**

A total of 49 cases submitted FCPs for evaluation of oncogenic driver alterations and were assessed using Amoy 9‐in‐1 and next‐generation sequencing (NGS) assays. The success rates of DNA and RNA analyses using the Amoy 9‐in‐1 were both 100%, compared with 86% and 45%, respectively, using NGS assays. Oncogenic driver alterations were detected in 27 (55%) and 23 (47%) patients using Amoy 9‐in‐1 and NGS, respectively. No inconsistent results were observed among 19 cases in which both assays showed successful detection. In the remaining 30 cases, 10 had inconsistent results: nine oncogenic driver alterations (3 *MET*, 2 *ALK*, 2 *ROS1*, and 2 *KRAS*) were detectable only in Amoy 9‐in‐1, and one epidermal growth factor receptor (*EGFR*) mutation was detectable only in NGS.

**Conclusion:**

FCPs can be successfully used in the AmoyDx PLC panel, with higher success rate compared with the NGS assay. The AmoyDx PLC panel may be an option in cases when insufficient tissue sample is available for the NGS assay.

## Graphical abstract

We retrospectively evaluated the performance of frozen cell pellets from cytology specimens (FCPs) in the Amoy 9‐in‐1 assay. The success rates of DNA and RNA analyses were both 100% in Amoy 9‐in‐1 assay, compared with 86% and 45%, using NGS assay. Although the coverage of Amoy 9‐in‐1 is limited compared to NGS assays, the Amoy using FCPs can be a reasonable option for the assessment of driver alteration, especially in cases where sufficient tissue specimen for the NGS assay is unavailable.

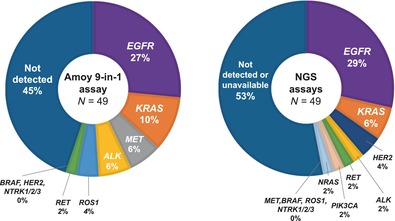

## INTRODUCTION

The treatment of non‐small cell lung cancer (NSCLC) improved significantly after the discovery of oncogenic driver alterations. Among them, molecular‐targeted drugs for NSCLC with oncogenic driver alterations have drastically improved clinical outcomes in recent decades, and the numbers of novel drugs are increasing.^1^, ^2^ Accordingly, various methods to detect oncogenic driver alterations, such as real‐time polymerase chain reaction (PCR) and next‐generation sequencing (NGS) assays, have been developed and are routinely used in clinical practice.^3^, ^4^, ^5^

The Amoy 9‐in‐1 assay (Amoy Diagnostics) is a real‐time multiplex PCR assay for qualitative detection of alterations in nine genes; *EGFR*, *ALK*, *ROS1*, *BRAF*, *MET*, *RET*, *KRAS*, *HER2*, and *NTRK*. Following the positive outcomes of the Amoy 9‐in‐1 assay in a multicenter prospective nationwide genomic screening system for lung cancer study (LC‐SCRUM‐Asia), the AmoyDx Pan Llng cancer PCR panel (AmoyDx PLC panel) has recently been approved in Japan as a companion diagnostic (CDx) tool for multiple molecular‐targeted drugs in patients with NSCLC.^6^ The AmoyDx PLC panel is expected to have a short turnaround time (TAT) with sensitivity equivalent to previously approved NGS assays, making it one of the primary assays currently used for detecting multiple oncogenic driver alterations in Japan, China, and Europe.^6^, ^7^ However, the AmoyDx PLC panel requires 7–10 slides of 5 μm formalin‐fixed paraffin‐embedded (FFPE) tissue samples for analysis. Although the amount of required specimen is smaller than that for NGS, it can be a significant obstacle for successful assessment.

The major methods for obtaining specimens are transbronchial biopsy (TBB), endobronchial ultrasound sonography transbronchial needle aspiration biopsy (EBUS‐TBNA), and percutaneous core needle biopsy (CNB). Despite the development of biopsy devices and techniques, obtaining a sufficient amount of sample material, especially from peripheral nodules or small tumors, remains challenging.^8^ As an alternative, cytology specimens, such as fine needle aspiration supernatant or pleural fluid, reportedly can be used with a high success rate of 80%–90% in NGS assays.^9^, ^10^, ^11^, ^12^, ^13^ Although these reports provide the possibility to overcome the technical limitations of tissue biopsy, the suitability of frozen cell pellets from cytology specimens (FCPs) for the AmoyDx PLC panel is unknown. Therefore, we aimed to evaluate the performance of the Amoy 9‐in‐1 assay using FCPs in patients with NSCLC.

## METHODS

### Patients

Our primary objective was to determine the success rate of Amoy 9‐in‐1 assay when using FCPs as a specimen. In addition, we evaluated the concordance with NGS using the same specimen, to determine the availability of Amoy 9‐in‐1 using FCPs in the real‐world practice.

In order to achieve these objectives, we retrospectively collected data of patients with NSCLC enrolled in LC‐SCRUM‐Asia (UMIN ID: 000036871) from the Shizuoka Cancer Center between September 2019 and May 2021. LC‐SCRUM‐Asia allowed both fresh frozen tissues and FCPs from cytology specimens. We evaluated patients in whom FCPs from cytology specimens had been submitted. Clinical data, including age, sex, smoking history, clinical stage, histology, biopsy method, biopsy site, and median time required for pathological diagnosis, were collected from the medical records. The genetic results were collected from the LC‐SCRUM‐Asia reports. LC‐SCRUM‐Asia was approved by the institutional review boards of the Shizuoka Cancer Center and National Cancer Center. The study was conducted in accordance with the principles of the Declaration of Helsinki. Written informed consent was obtained from all the patients.

### Methods of specimen sampling

Cytology specimens were obtained through TBB, EBUS‐TBNA, or CNB. Every performed biopsy involved both sampling and washing. For TBB, the biopsy forceps were rinsed with 20 mL saline for a few seconds after each biopsy. In EBUS‐TBNA and CNB, the needle was washed with saline solution after each puncture. TBB was usually performed around five times, with or without several rounds of bronchial brushing. EBUS‐TBNA and CNB were performed at least twice. After the biopsies, cytological diagnosis was performed using half of the saline specimens, and the remaining were allocated as specimens for LC‐SCRUM‐Asia. The specimens were centrifuged at 1630 *g* for 5 min; the supernatants were subsequently removed, and the cell pellets were stored at −80°C without DNA/RNA‐stabilizing solutions. The obtained tissue and cytology specimens were quickly sent to the pathology department, where they diagnosed and submitted the report to the physicians. Tissue samples were processed as FFPE and then stained with hematoxylin and eosin (H&E) or other immunostaining if necessary. Cytology samples were generally fixed by 95% ethanol and then Papanicolaou‐stained before diagnosis. Malignancy of the cytology specimens was confirmed by two pathologists, who commented on tumor positivity. The physician decided on enrollment into LC‐SCRUM‐Asia according to the pathology report.

### Analysis in LC‐SCRUM‐Asia

After enrollment in LC‐SCRUM‐Asia, FCPs were sent to a Clinical Laboratory improvement amendments‐certified clinical laboratory (SRL Inc., Tokyo, Japan). The submitted FCP specimens were eluted for DNA/RNA in 50 μL of dedicated solution and extracted for DNA and RNA using an Allprep DNA RNA mini kit, a nucleic acid extraction kit. Based on the required amount of DNA/RNA of 45 ng, a maximum amount of 22.5 μL was used for a single assay. The yield of DNA and RNA was quantified by the NanoDrop (Thermo Fisher Scientific) or Qubit fluorometric assay (Thermo Fisher Scientific).^14^ If analysis was unable to be performed due to lack of the amount of DNA/RNA, it was reported as “insufficient”.

The specimens were then analyzed for driver alterations via the Amoy 9‐in‐1 assay and an NGS assay (either the Oncomine comprehensive assay version 3 [OCA, Thermo Fisher Scientific] or the Oncomine precision assay [OPA, Thermo Fisher Scientific]), depending on the time of submission (OCA; from September 2019 to December 2020, OPA; from January 2021 to May 2021).

In the Amoy 9‐in‐1 assay, driver alterations were detected by DNA‐based sequences (*EGFR*, *BRAF*, *KRAS*, and *HER2*) and RNA‐based sequences (*ALK*, *ROS1*, *MET*, *RET*, and *NTRK*). In contrast, NGS panels are designed to cover typical cancer genes in addition to those without targetable therapy; the OCA detects 161 of the most relevant cancer genes, whereas the OPA detects the most prevalent and potentially relevant cancer driver variants across 50 genes. In addition to nine targetable driver alterations, the results of OCA include *FGFR1/2/3/4*, *NRG1*, *AKT1*, *ERBB2*, *HRAS*, *NRAS*, *PIK3CA*, and OPA include *AKT1/2/3*, *AR*, *ARAF*, CD274, *CDK4*, *CDKN2A*, *CHEK2*, *CTNNB1*, *ERBB2/3/4*, *ESR1*, *FGFR1/2/3/4*, *FLT3*, *GNA11*, *GNAQ*, *GNAS*, *HRAS*, *IDH1/2*, *KIT*, *MAP2K1/2*, *MTOR*, *NRAS*, *PDGFRA*, *PIK3CA*, *PTEN*, *RAF1*, *SMO*, and *TP53*. Both the NGS and Amoy 9‐in‐1 assays were performed using the same specimen.

### Statistical analysis

Statistical analyses were performed using EZR software version 1.6‐3 (Saitama Medical Center, Jichi Medical University), which adds frequently used biostatistical functions to an original R commander.^15^ The median time required for pathological diagnosis were compared using the Mann–Whitney U test, and *p*‐values of <0.05 were considered statistically significant.

## RESULTS

### Patient characteristics and specimens

A total of 3878 patients were enrolled in LC‐SCRUM‐Asia, of which 70 were enrolled from the Shizuoka Cancer center. Among the 70 patients, 49 cases submitted FCPs from cytology specimens for evaluation of oncogenic driver alterations and were assessed using the Amoy 9‐in‐1 and NGS assays.

Patient characteristics and specimens are listed in Table 1. Among the patients, the median age was 67 years (range: 33–83 years) of which 55% were male, 71% had a history of smoking, 82% were diagnosed with clinical stage IV, and 94% were diagnosed with adenocarcinoma. The FCP specimens were collected using TBB bronchial brushing (69%), EBUS‐TBNA (20%), or CNB (10%). The biopsy sites were located primarily in the lung (78%), followed by the lymph node (16%), liver (4%), and soft tissue (2%). The median time required for pathological diagnosis of cytology specimens was 1 day (range: 0–6 days), which was significantly shorter than that of FFPE specimens (median: 6 days; range: 2–15 days, *p* < 0.001).

**TABLE 1:** Characteristics of the patients and submitted specimens (*N* = 49).

Characteristics		Patients	
		*N*	%
Age, years	Median	67	
	Range	33–83	
Sex	Male	27	55
	Female	22	45
Smoking history	Nonsmoker	14	29
	Smoker	35	71
Clinical stage	III	9	18
	IV	40	82
Histology	Adenocarcinoma	46	94
	NOS	3	6
Biopsy method	TBB	34	69
	EBUS‐TBNA	10	20
	CNB	5	10
Biopsy site	Lung	38	78
	Lymph node	8	16
	Liver	2	4
	Soft tissue	1	2

Abbreviations: CNB, core needle biopsy; EBUS‐TBNA, endobronchial ultrasound‐guided transbronchial needle aspiration; NOS, not otherwise specified; TBB, transbronchial biopsy.

### Assessment in LC‐SCRUM‐Asia

**FIGURE 1:**
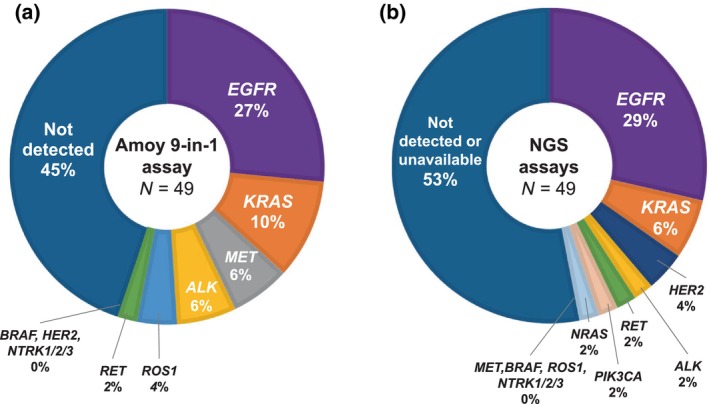
Distribution of oncogenic driver alterations detected by the Amoy 9‐in‐1 assay (a) and the next‐generation sequencing (NGS) assays (b) in patients with non‐small cell lung cancer.

The success rates of DNA and RNA analyses using the Amoy 9‐in‐1 assay were both 100%, regardless of the biopsy method. Oncogenic driver alterations were detected in 27 patients (55%) using the Amoy 9‐in‐1 assay: 13 *EGFR* mutations (27%), five *KRAS* mutations (10%), three *MET* mutations (6%), three *ALK* fusions (6%), two *ROS1* fusions (4%), one *RET* fusion (2%), and no *BRAF*, *HER2*, nor *NTRK* fusions (0%) (Figure 1a). On the other hand, the success rates of DNA and RNA analyses in NGS assays were 86% and 45%, respectively. In each biopsy method, the respective success rates of DNA and RNA analyses were 91% and 38% for TBB, 90% and 70% for EBUS‐TBNA, and 40% and 0% for CNB. Oncogenic driver alterations were detected in 23 patients (47%) using NGS assays: 14 *EGFR* mutations (29%), three *KRAS* mutations (6%), two *HER2* amplifications (4%), one *ALK* fusion (2%), one *RET* fusion (2%), one *NRAS* mutation (2%), and one *PIK3CA* mutation (2%) (Figure 1b).

**FIGURE 2:**
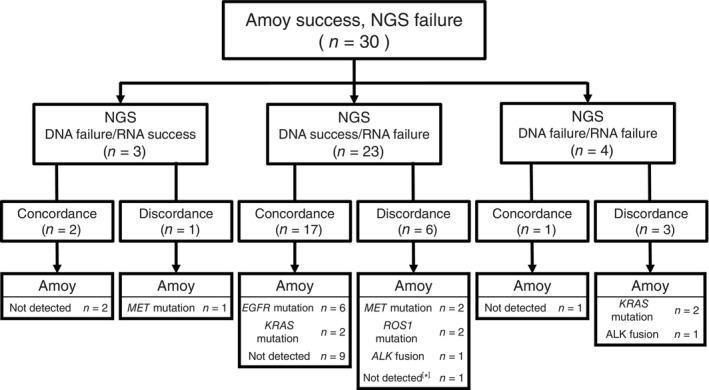
Patient flow of all next‐generation sequencing (NGS) failure cases (*n* = 30). The results of failure cases are classified to three categories: DNA success but RNA failure, DNA failure but RNA success, and DNA/RNA failure. In each category, the concordance and discordance with the results from the Amoy 9‐in‐1 assay are shown. In [*] case, *E709 delE709_T710insD* was detectable via NGS, which was not detected via Amoy.

A total of 19 cases had both DNA and RNA analysis successfully performed via NGS assays, and no discordance was observed in the results compared with the Amoy 9‐in‐1 assay. In the remaining 30 cases, 10 had inconsistent results: nine oncogenic driver alterations (3 *MET* mutations, 2 *ALK* fusions, 2 *ROS1* fusions, and 2 *KRAS* mutations) were detectable only via Amoy 9‐in‐1, and one *EGFR* mutation (*E709 delE709_T710insD*) was detectable only via NGS (Figure 2). The discordance in the detection of oncogenic driver alterations between the Amoy 9‐in‐1 and the NGS assays are listed in Table 2. In nine of the cases for which the oncogenic driver alterations were detected only by Amoy 9‐in‐1, seven cases were able to have access to treatment with molecular‐targeted drugs.

**TABLE 2:** Cases with discordant results between Amoy 9‐in‐1 and NGS (*N* = 10).

Case	Age	Sex	Histology	Biopsy method	Amoy 9‐in‐1	NGS	CDx confirmation	Molecular‐targeted therapy
1	76	F	Ad	TBB	*MET* Ex14 skip	(−)	Archer MET	Tepotinib
2	65	M	Ad	TBB	*MET* Ex14 skip	(−)	Archer MET	Tepotinib
3	74	M	Ad	TBB	*MET* Ex14 skip	(−)	NA	MET inhibitor Clinical trial
4	56	F	Ad	TBB	*ALK* fusion	(−)	Oncomine	Alectinib
5	44	F	Ad	CNB	*ALK* fusion	(−)	ALK IHC	Alectinib
6	66	F	Ad	TBB	*ROS1* fusion	(−)	Amoy ROS1	ROS1 inhibitor Clinical trial
7	76	F	Ad	TBB	*ROS1* fusion	(−)	Amoy ROS1	ROS1 inhibitor Clinical trial
8	70	F	Ad	CNB	*KRAS* others	(−)	NA	NA
9	75	M	Ad	CNB	*KRAS* others	(−)	NA	NA
10	77	M	Ad	TBB	(−)	*EGFR* mutation E709_T710 > D	NA	NA

Abbreviations: Ad, adenocarcinoma; Amoy ROS1, OncoGuide AmoyDx ROS1 gene fusions detection kit; Archer MET, Archer MET companion diagnostic system; CDx, companion diagnostic; CNB, core needle biopsy; EGFR, epidermal growth factor receptor; KRAS others, *KRAS* mutations besides G12C; NA, not applicable; NGS, next‐generation sequencing (Oncomine comprehensive assay [OCA] or Oncomine precision assay [OPA]); Oncomine, Oncomine Dx target test; TBB, transbronchial biopsy.

## DISCUSSION

In this study, we aimed to evaluate the performance of the Amoy 9‐in‐1 assay using FCPs in patients with NSCLC. Our study demonstrated that the use of FCPs in the Amoy 9‐in‐1 assay resulted in sufficient success rates, indicating the suitability of cytology specimens in clinical practice. Furthermore, this success rate was higher than that for NGS assays, showing that the Amoy 9‐in‐1 assay may be more appropriate than NGS assays when cytology specimens are used. To the best of our knowledge, this is the first study to evaluate the success rate of Amoy 9‐in‐1 assay when using FCPs as a specimen.

Normally when detecting driver alteration in multiple PCR or NGS assays in clinical practice, a sufficient amount of tissue specimen is required. The main reason for this is that, compared with FFPE specimens obtained via surgery, cytology and biopsy specimens are reportedly more vulnerable and contain fewer tumor‐related DNA and RNA molecules, leading to a harsh condition.^7^, ^11^, ^12^ Furuya et al. demonstrated the use of bronchial brushing cytology specimens with the NGS assay and noted that the success rate of RNA analysis for such specimens was 80.4%, relatively lower than those of surgical biopsy FFPE samples.^14^ Likewise, our study showed a low success rate of 45% for RNA in NGS. On the other hand, despite using the same cytology specimen as in the NGS assay, we demonstrated a 100% success rate for both DNA and RNA in the Amoy 9‐in‐1 assay using FCPs, including several specimens that were unsuitable for the NGS assay. This contrast in the success rates of the Amoy 9‐in‐1 and NGS assays may be attributable to the difference in assessment method. Unlike the Amoy 9‐in‐1, which is a PCR panel‐based assay, the NGS panel‐based assays require a more complex testing process with a higher quantity or quality of DNA and RNA from specimens.^7^, ^16^ Also, previous reports and manufacturers' protocol have estimated the limit of detection (allele frequency %) in the AmoyDx PLC panel to be 1%–5%, which is at least equivalent to those of Oncomine Dx target test, 6%–10%.^17^, ^18^ Although the limit of detection in each assay is not strictly comparable due to varying conditions, Amoy 9‐in‐1 could be a reasonable option for the assessment of driver alteration, where novel high‐sensitive assays such as droplet digital PCR are currently unavailable in a clinical practice.

Nevertheless, we should note that although Amoy‐9‐in‐1 covers an adequate number of variants, its coverage is limited compared with NGS assays.^19^ For example, in our study, Amoy 9‐in‐1 could not detect *EGFR E709 delE709_T710insD*, whereas this mutation was detectable in the NGS assay. Owing to this limitation, assessment using NGS assays may be preferred if a sufficient quantity or quality of specimen can be obtained via biopsy, although these variants are rarely detected. Japanese medical insurance only allows these panels to be used once, making a high success rate even more crucial for patients regardless of the difficulty in sampling specimens. Accordingly, the Amoy 9‐in‐1 assay may be an ideal method for analyzing oncogenic driver alterations in cases for which collecting a sufficient amount of specimen is difficult. Of note, nine cases of oncogenic driver alterations were detected only via Amoy 9‐in‐1 in this study; seven of these were successfully treated with molecular‐targeted drugs. These cases could have lost their treatment option if they were assessed only by NGS assays.

We believe there are several advantages in using FCPs in the AmoyDx PLC panel. First, the pathological diagnosis of cytology is usually faster than that of histology. The turn‐around time of the AmoyDx PLC panel is reportedly significantly shorter than that of the OCA and OPA.^7^ In clinical settings where early diagnosis and treatment are extremely important, the AmoyDx PLC panel may be one of the most promising methods for the rapid detection of oncogenic driver alterations. Second, in contrast to FFPE, FCPs can be retrieved via bronchial brushing, which is less invasive and reduces the risk of bleeding during bronchoscopy, making it especially beneficial for patients at risk of hemorrhage.^20^ Finally, FCPs are relatively easier to obtain compared to FFPE. When using tissue samples, the multiplex Amoy Dx PLC panel and NGS usually requires at least 7–10 slides of 5 μm FFPE, which could be a severe requirement especially when the target nodule is particularly small and/or is technically difficult to collect a sufficient amount of specimen. Notably, the results of neoadjuvant trials targeting early‐stage NSCLC with oncogenic driver alterations may affect clinical practice in the near future, generating a greater need for collecting specimens from smaller peripheral lesions.^21^

Nonetheless, the present study also had some limitations. First, it was a single‐center, retrospective study with a relatively small number of patients. Second, since Amoy 9‐in‐1 is a precommercial assay of the AmoyDx PLC panel, it should be noted that both assays are not exactly the same; for example, in the threshold for mutation positivity. Third, we could not confirm the results of the Amoy 9‐in‐1 assay using FCPs to other CDx assays such as the AmoyDx PLC panel using FFPE, or FoundationOne CDx. Also, the actual yield of DNA/RNA genes and variant allele fractions in the specimens were not available.

In conclusion, we demonstrated the successful use of FCPs for the Amoy 9‐in‐1 assay, with a high concordance rate with NGS assays. Although the AmoyDx PLC panel can offer limited variants compared with NGS assay, it is expected to have a higher success rate than NGS assay when cytology specimens are used. The AmoyDx PLC panel using FCPs can be an alternative method for analyzing oncogenic driver alterations in patients with NSCLC for whom sufficient amount of tissue specimen for the NGS assay cannot be obtained.

## AUTHOR CONTRIBUTIONS

**Hiroaki Kodama:** Writing—original draft. **Haruyasu Murakami:** Conceptualization, project administration, investigation, writing—review and editing. **Nobuaki Mamesaya:** Resources, writing—review and editing. **Haruki Kobayashi:** Resources, writing—review and editing. **Shota Omori:** Resources, writing—review and editing. **Kazushige Wakuda:** Resources, writing—review and editing. **Ryo Ko:** Resources, writing—review and editing. **Akira Ono:** Resources, writing—review and editing. **Hirotsugu Kenmotsu:** Resources, writing—review and editing. **Tateaki Naito:** Resources, writing—review and editing. **Shingo Matsumoto:** Resources, writing—review and editing. **Koichi Goto:** Resources, writing—review and editing. **Tetsuo Shimizu:** Writing—review and editing. **Yasuhiro Gon:** Writing—review and editing. **Toshiaki Takahashi:** Resources, writing—review and editing.

## FUNDING INFORMATION

This research did not receive any specific grant from funding agencies in the public, commercial, or not‐for‐profit sectors.

## CONFLICT OF INTEREST STATEMENT

LC‐SCRUM‐Asia was supported by Amgen, Astellas, AstraZeneca, Boehringer‐Ingelheim, Bristol‐Myers, Chugai, Daiichi‐Sankyo, Eisai, Eli Lilly, Janssen, Kyowa Kirin, Merck Serono, MBL, MSD, Novartis, Ono, Pfizer, Sumitomo Dainippon, Taiho, and Takeda. H.K reports personal fees from Chugai pharma, Daiichi Sankyo, and Novartis Pharma K.K. outside the submitted work. H.M reports grants and personal fees from AstraZeneca, Daiichi Sankyo, Chugai pharma, Taiho Pharmaceutical, grants from IQvia, Bayer, personal fees from Takeda, Ono Pharmaceutical, Bristol‐Myers Squibb Japan, MSD, Pfizer, Novartis, Lilly Japan, Taiho Pharmaceutical, Eisai, Amgen, and Nihonkayaku, and participation on a data safety monitoring board or advisory board, outside the submitted work. N.M reports receiving grants and personal fees from Boehringer Ingelheim, personal fees from Chugai Pharmaceutical Co., Ltd., Taiho Pharmaceuticals, MSD K.K., AstraZeneca K.K., and Ono Pharmaceutical Co., Ltd., outside the submitted work. H.K reports receiving personal fees from Eli Lilly K.K., Novartis Pharma K.K., Taiho Pharmaceutical., AstraZeneca K.K., Chugai Pharmaceutical Co., Ltd., Ono Pharmaceutical Co., Ltd., Bristol‐Myers Squibb Company, and Daiichi Sankyo Co., outside the submitted work. S.O reports grants and personal fees from Daiichi Sankyo, personal fees from Ono Pharmaceutical, Japan Eli Lilly Co., Ltd., Taiho Pharmaceutical Co., Ltd., Chugai Pharmaceutical Co., Ltd., Amgen K.K., and AstraZeneca K.K., Kyowa Kirin Co., Ltd., MSD K.K., Bristol Myers Squibb K.K., and Novartis Pharma K.K outside the submitted work. K.W reports receiving grants and personal fees from Chugai Pharmaceutical Co., Ltd., AstraZeneca K.K., MSD K.K., and Daiichi Sankyo Co., Ltd., grants from Novartis Pharma K.K., AbbVie, AMGEN, Dizal Pharma, personal fees from Taiho Pharmaceutical, Boehringer Ingelheim, Eli Lilly K.K, Ono Pharmaceutical, Janssen Pharmaceutical K.K., Takeda Pharmaceutical, and Nihon Kayaku, outside the submitted work. A.O reports receiving personal fees from AstraZeneca K.K., Chugai Pharmaceutical Co., Ltd., Ono Pharmaceutical Co., Ltd., and Indica labs, outside the submitted work. H.K reports receiving, grants and personal fees from AstraZeneca K.K., Novartis Pharma K.K., Ono Pharmaceutical Co, Ltd., and Eli Lilly K.K., personal fees from AMGEN, Bayer, Boehringer Ingelheim, Bristol‐Myers Squibb, Chugai Pharmaceutical Co., Daiichi‐Sankyo Co., Ltd., Kyowa Hakko Kirin Co., Ltd., Taiho Pharmaceuticals, Takeda Pharmaceutical Co., Ltd, Merck Biopharma Co., Ltd., MSD K.K, and Pfizer, and grants from Loxo Oncology, outside the submitted work. T.N reports receiving grants from Otsuka Pharmaceutical K.K., and Japan Agency for Medical Research and Development (AMED), personal fees from Ono Pharmaceutical Co., Ltd., and Helsinn Healthcare SA, outside the submitted work. S.M reports grants and personal fees from Lilly Japan, Novartis, and Merck Biopharma, grants from Chugai pharma and MSD, and personal fees from AstraZeneca outside the submitted work. K.G reports support for the present manuscript from Amoy Diagnostics, Riken Genesis, Pfizer Japan, Novartis Pharma, AstraZeneca, Chugai Pharmaceutical, Takeda Pharmaceutical, Merck Biopharma, and Boehringer Ingelheim Japan, grants and personal fees from Amgen K.K., AstraZeneca K.K., Bristol‐Myers Squibb K.K., Chugai Pharmaceutical Co., Ltd., Daiichi Sankyo Co., Ltd, Eisai Co., Ltd., Eli Lilly Japan K.K., Janssen Pharmaceutical K.K., Merck Biopharma Co., Novartis Pharma K.K., Ono Pharmaceutical Co., Ltd., Taiho Pharmaceutical Co., Ltd., Takeda Pharmaceutical Co., Ltd., grants from Amgen Inc., AbbVie GK, AnHeart Therapeutics Inc., Bayer Yakuhin, Ltd., Boehringer Ingelheim Japan, Inc., Blueprint Medicines Corporation., Craif Inc., Guardant Health Asia, Middle East & Africa, Inc, Haihe Biopharma Co., Ltd., Ignyta, Inc., Kyowa Kirin Co., Ltd., Life Technologies Japan Ltd., Loxo Oncology, Inc., Lunit Inc., Medical and Biological Laboratories Co. Ltd., Merus N.V., MSD K.K., Pfizer R&D Japan G.K., Precision Medicine Asia Co., Ltd., Riken Genesis Co. Ltd., Sumitomo Pharma Co., Ltd., Spectrum Pharmaceuticals, Inc., Sysmex Corporation., Turning Point Therapeutics, Inc., personal fees from Amoy Diagnosties Co., Ltd., Bayer U.S, Guardant Health Japan Corp., iTeos Therapeutics Inc., Thermo Fisher Scientific K.K., Ltd., Nippon Kayaku Co., Ltd., Pharma Mar, S.A., Riken Genesis Co. Ltd., and participation on a Data safety monitoring Board or Advisory Board in Amgen Inc, Amgen K.K, AstraZeneca K.K, Bayer HealthCare Pharmaceuticals Inc., Bristol‐Myers Squibb K.K, Daiichi Sankyo Co., Ltd, Eli Lilly Japan K.K., GlaxoSmithKline K.K, Haihe Biopharma Co., Ltd, Janssen Pharmaceutical K.K, Syneos Health Clinical K.K, T.S reports grants and personal fees from Chugai Pharmaceutical Co., Ltd. and Ono Pharmaceutical Co., Ltd., grants from Eli Lilly K.K., and personal fees from MSD, AstraZeneca K.K., and Taiho Pharmaceuticals, outside the submitted work. T.T reports receiving grants and personal fees from AstraZeneca K.K., Chugai Pharmaceutical Co., Ltd., Eli Lilly Japan K.K., MSD, Pfizer Japan Inc., and Amgen Inc., grants from Merck Biopharma CO., LTD, Janssen Pharmaceutical K.K., and AnHeart Therapeutics Inc., personal fees from BMS Japan, Takeda Pharmaceuticals Co., Novartis You, and Ono Pharmaceutical Co., Ltd., outside the submitted work. The other authors have no conflicts of interest to disclose.

## Data Availability

The data that support the findings of this study are available on request from the corresponding author. The data are not publicly available due to privacy or ethical restrictions.
